# Autoantibodies against zinc transporter 8 are related to age and metabolic state in patients with newly diagnosed autoimmune diabetes

**DOI:** 10.1007/s00592-017-1091-x

**Published:** 2018-01-11

**Authors:** Elżbieta Niechciał, Anita Rogowicz-Frontczak, Stanisław Piłaciński, Marta Fichna, Bogda Skowrońska, Piotr Fichna, Dorota Zozulińska-Ziółkiewicz

**Affiliations:** 1 Department of Pediatric Diabetes and Obesity, Poznan University of Medical Sciences, Szpitalna Street 27/33, 60-572 Poznan, Poland; 2 Department of Internal Medicine and Diabetology, Poznan University of Medical Sciences, Mickiewicza Street 2, 60-101 Poznan, Poland; 3 Department of Endocrinology, Metabolism and Internal Medicine, Poznan University of Medical Sciences, Przybyszewskiego Street 49, 60-101 Poznan, Poland; 4 Institute of Human Genetics, Polish Academy of Sciences, Strzeszyńska Street 32, 60-479 Poznan, Poland

**Keywords:** Childhood type 1 diabetes, Adult type 1 diabetes, Autoimmune diabetes diagnosis, Autoimmunity, Diabetes-related autoantibodies, Autoantibodies to zinc transporter-8

## Abstract

**Aims:**

To assess the prevalence of ZnT8-ab and its correlation to other autoimmune markers and diabetic ketoacidosis occurrence in children and adults with T1DM onset.

**Methods:**

The study included 367 patients (218 children; 149 adults) at the T1DM onset. Selected diabetes-related autoantibodies such as GAD-ab, IA2-ab, ZnT8-ab were tested before the initiation of insulin therapy. Diabetic ketoacidosis was defined as glucose concentration > 13.9 mmol/l, pH < 7.30, concentration of HCO_3_ < 15 mmol/l, presence of ketone bodies in the blood and urine.

**Results:**

The autoantibodies pattern differs in both study groups. Children were mostly positive for two (37.8%) and three (49.5%) autoantibodies, whereas adults for one (32.2%) and two (30.7%). The most frequently detected autoantibodies in youth were ZnT8-ab (81.1%) and IA2-ab (80.7%), while in adults GAD-ab (74.8%). ZnT8-ab (*p* < 0.0001) titers were significantly higher in children, but adults had higher titer of GAD-ab (*p* < 0.0001) and IA2-ab (*p* < 0.0001). Children developed more frequently diabetic ketoacidosis (28.4 vs. 10.7%, *p* = 0.0002). ZnT8-ab (*p* = 0.002) and IA2-ab (*p* = 0.008) were reported mostly in individuals with ketoacidosis. A correlation between the number of positive antibodies and the severity of ketoacidosis was observed (*R*_s_ − 0.129 *p* = 0.014). ZnT8-ab were associated with a greater risk of ketoacidosis independent of gender, age group and the autoantibodies number [OR = 2.44 (95% CI 1.0–5.94), *p* = 0.04].

**Conclusions:**

Children are at greater risk of ketoacidosis at the diagnosis of diabetes. ZnT8-ab and IA2-ab are commonly detected in children, while adults have frequently higher titer of GAD-ab. ZnT8-ab are associated with more acute diabetes onset.

**Electronic supplementary material:**

The online version of this article (10.1007/s00592-017-1091-x) contains supplementary material, which is available to authorized users.

## Introduction

Type 1 diabetes (T1DM) results from immune dysregulation in which the immune response is specifically directed against pancreatic β-cells. Despite numerous investigations on T1DM origin, the mechanism of this process remains unclear and T1DM is considered as a multifactorial disease. To this point, no single factor inducing the destructive process has been identified. Genetic susceptibility and many environmental agents, including gluten containing food, play a role in disease origin [1–6].

T1DM is mostly diagnosed in children, but it can emerge in adult individuals as well [7]. Diabetic ketoacidosis (DKA) can be a first manifestation of T1DM at any age. Some individuals may retain sufficient residual β-cells function to prevent DKA for many years. The loss of the pancreatic islet β-cells mass seems to be more rapid in children than in adults [8].

The presence of autoantibodies is useful for diagnostic support of T1DM. These comprise islet-cell antibodies (ICA), glutamic acid decarboxylase autoantibodies (GAD-ab), protein tyrosine phosphatase autoantibodies (IA2-ab) and insulin autoantibodies (IAA). The majority of patients at T1DM onset are positive for at least one of above-mentioned antibodies [9]. GAD-ab is the most commonly detected autoantibody, found in 50–80% individuals [10, 11]. While IAA occurs in very young children and displays a strong inverse correlation with patient’s age at the time of T1DM diagnosis [12, 13], IAA is found in 40–70% children at diagnosis [4, 6]. IA2-ab is reported in 32–75% patients at T1DM onset [14, 15]. GAD-ab and ICA are also significant in latent autoimmune diabetes of adults (LADA) diagnosis [16, 17].

Moreover, diabetes-related autoantibodies might be used as progression markers to T1DM. Velluzii et al. [18] reported that throughout the 10-year study period, the risk of having T1DM was 55.3 times higher in children with any single positive autoantibody and rose by 14.5 times in individuals with any double autoantibodies. Kohler et al. observed a positive association between the time to development of T1DM and the seroconversion occurrence. In detail, a positive association was seen up to 54 months for IAA, between 6 and 36 months for IA2-ab and up to 18 months for GADA-ab. [19]

Recently, ZnT8-ab have been identified as a novel target of T1DM autoimmunity. ZnT8 is a 369-amino acid transmembrane protein, encoded by the *SLC30A8* gene at the chromosome 8q14.11 [20, 21]. ZnT8-ab are found in 66–80% Caucasian patients at disease diagnosis. Additionally, based on the study performed by Wenzlau et al., they could be detectable in 26% individuals with T1DM previously classified as autoantibody negative. Therefore, the autoimmunity detection rate could increase up to 98% if the measurement of ZnT8-ab was added (20). Nevertheless, ZnT8-ab prevalence varies in different population. For example, ZnT8-ab were present only in 28% of the Japanese patients at T1DM onset [22].

Although, ZnT8-ab are reported in the majority of T1DM patients prior to and at clinical diagnosis, the relationship between the presence of ZnT8-ab and severity of the initial clinical manifestation remains ambiguous. This study aims to assess and compare the prevalence of ZnT8-ab and its correlation to other autoimmune markers and DKA at the presentation of T1DM in children and adults.

## Methods

### Patients

We studied the prevalence of GAD-ab, IA2-ab and ZnT8-ab in children and adults with newly diagnosed T1DM between 2010 and 2014. Moreover, IAA were measured in pediatric group, and ICA testing was performed in all patients with adult onset of diabetes. All participants were an ethnically homogenous population from Poland. The cohort comprised 367 European Caucasian patients, including: 218 children [girls: 95; boys: 123; median age: 9 years (interquartile range: 6–13 years)] and 149 adults [women: 78; men: 71; median age: 34 years (interquartile range: 27–43) years)], admitted to the Department of Pediatric Diabetes and Obesity, and to the Department of Internal Medicine and Diabetology of the Poznan University of Medical Sciences. The study was approved by the Poznan University of Medical Sciences Ethics Committee (decisions No 1623/05, No 739/09 and No 960/12). Informed consent was obtained from all individual participants included in the study.

### Diabetes diagnosis

Diabetes was diagnosed based upon WHO criteria, including fasting plasma glucose ≥ 7.0 mmol/l, 2-h postprandial plasma glucose ≥ 11.1 mmol/l, during an oral glucose tolerance test, classic symptoms of hyperglycemia or hyperglycemic crisis, as well as random plasma glucose concentration of ≥ 11.1 mmol/l. The first day of insulin administration was at T1DM diagnosis.

### Clinical and laboratory data

Standard laboratory tests, including blood glucose, capillary blood gases and blood or urinary ketones, were performed in every patient. DKA was defined as blood glucose concentration > 13.9 mmol/l, blood pH < 7.30, concentration of HCO_3_ < 15 mmol/l, detection of ketones in the urine or elevated ketones in the serum, anion gap > 12. It was considered as mild, moderate and severe if pH was < 7.3 ≥ 7.2, < 7.2 ≥ 7.1 and < 7.1, respectively. Level of C-peptide was assessed by radioimmunoassay [C-PEP II-RIA-CT, DIA source Immunoassay, S.A, Belgium]. Glycated hemoglobin (HbA1c) value was evaluated by Chemiluminescent Microparticlr Immunoassey [HbA1C ARCHITECT System, Abbott Laboratories, U.S.A.]. To confirm autoimmune diabetes origin, typical autoantibodies were tested. The blood samples were collected on the day of diagnosis, before an initial insulin therapy, centrifuged to obtain serum and frozen until analyzed. GAD-ab and IA2-ab were measured by the RIA test kits, GAD-ab assay (positivity: > 1 U/ml) and IA-2-ab assay (positivity: > 1 U/ml), respectively [GAD and IA2 RIA. EUROIMMUN, Germany]. Level of ZnT8-ab was determined by the enzyme-linked immunosorbent assay [ElisaRSR ZnT8 Ab^™^, UK]. The upper limit of the normal range was 15 U/mL, according to the manufacturer’s recommendation. Although IAA were checked in children, these antibodies are less likely to be present in older individuals and hence were not screened in adults and are not considered in the study.

### Statistical analysis

The results are displayed as means and standard deviations (± SD), medians and interquartile ranges (IQR) or as numbers and percentages (%). Normality of the data distributions was tested using D’Agostino-Pearson test. We compared groups with different profiles of diabetes-associated autoantibodies using Student’s *t* test or Mann–Whitney *U* test for continuous variables and Fisher’s exact test for categorical variables. We calculated Pearson’s correlation coefficients to determine the association between selected continuous variables and the titer of diabetes-related autoantibodies.

We performed all tests at a significance level of 0.05 (two sided). Statistical analyses were carried out using Statistica version 10 (StatSoft Inc., Tulsa, OK, USA) and MedCalc version 17.5.3 (MedCalc Statistical Software, Ostend, Belgium).

## Results

Laboratory tests revealed that children demonstrated higher glucose concentration (24.8 ± 10.6 vs. 18.8 ± 8.1 mmol/l, *p* = 0.0017) and lower C-peptide level (0.4 ± 0.2 vs. 1.1 ± 0.7 pmol/ml, *p* < 0.0001) compared to adults. Accordingly, children were more frequently diagnosed with DKA (28.4 vs. 10.7%, *p* = 0.0002) (Table 1).

**Table 1:** Clinical features and autoantibodies spectrum in children and adults with newly diagnosed type 1 diabetes

Variable	Children (*n* = 218)	Adults (*n* = 149)	*p* value
Clinical characteristics			
Age (year) [IQR]	9.0 (6.0–13.0)	34.0 (27.0–43.0)	NA
Sex (F:M)	95:123	78:71	NS
DKA (%)	28.4	10.7	*p* = 0.0002
NGSP HbA_1C_ (%) [mmol/mol]	11.0 ± 2.1 [97 ± 18.5]	10.9 ± 2.7 [96 ± 23.8]	NS
Glycemia at admission (mmol/l)	24.8 ± 10.6	18.8 ± 8.1	*p* = 0.0017
C-peptide (pmol/l)	0.4 ± 0.2	1.1 ± 0.7	*p* < 0.0001
Autoantibodies			
Positive GAD-ab (%)	69.7	74.8	NS
GAD-ab titer (U/ml)	3.9 (0.5–17.7)	105.4 (8.9–545.8)	*p* < 0.0001
Positive IA2-ab (%)	80.7	44.0	*p* < 0.0001
IA2-ab titer (U/ml)	6.5 (1.5–19.3)	17.8 (7.0–111.7)	*p* < 0.0001
Positive ZnT8-ab (%)	81.1	34.8	*p* < 0.0001
ZnT8-ab titer (U/ml)	407.7 (35.5–524.5)	6.6 (0.0–157.9)	*p* < 0.0001
Number of positive autoantibodies			
1 antibody (%)	7.8	32.2	*p* < 0.0001
2 antibodies (%)	37.8	30.7	NS
3 antibodies (%)	49.5	18.1	*p* < 0.0001
Individuals positive for single antibody			
GAD-ab (%)	35.3	75.4	*p* = 0.013
IA2-ab (%)	29.4	12.1	NS
ZnT8-ab (%)	35.3	12.5	NS
Individuals positive for two antibodies			
GAD-ab + IA2-ab (%)	23.2	53.6	*p* = 0.0011
GAD-ab + ZnT8-ab (%)	23.2	41.5	NS
ZnT8-ab (%) + IA2-ab (%)	53.6	4.9	*p* < 0.0001

Data are expressed as means (±SD) or medians unless otherwise stated. C-peptide reference range: 0.59–1.54 pmol/ml

*IQR* interquartile range, *NA* non-assessed, *NS* non-significant

Two hundred and seven (95%) children and one hundred twenty-one (81.2%) adults were classified as at least one of three autoantibody positive at T1DM onset. The occurrence of multiple autoantibodies differed significantly between children and adults. The appearance of a unique autoantibody was rare in children, concerning only 7.8% individuals. Commonly, two (37.8%) or three (49.5%) diabetes-associated autoantibodies were detectable in children (*p* < 0.0001) compared to adults. Conversely, adults mostly presented with just one (32.2%) or two (30.7%) of the assessed antibodies. Only 18.1% adults displayed three positive autoantibodies (Table 1).

**Fig. 1:**
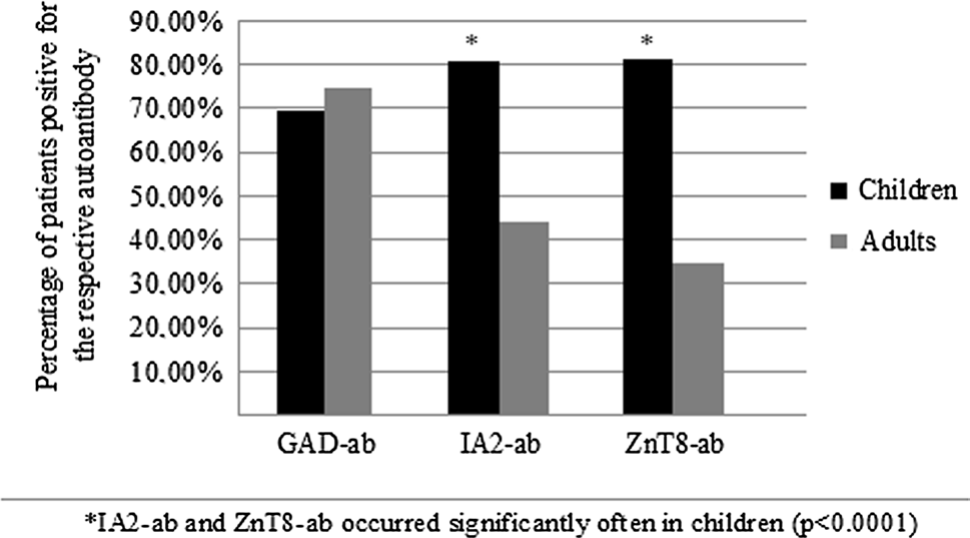
Autoantibodies spectrum in children and adults with newly diagnosed T1DM

**Fig. 2:**
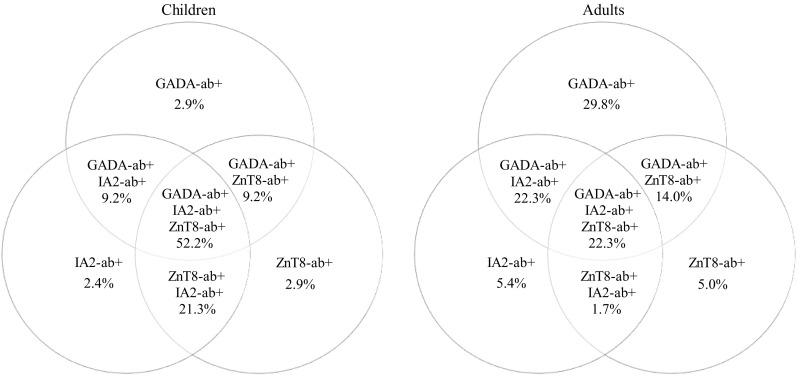
Venn diagram of autoantibody combinations in autoantibody-positive children and adults at T1DM onset

Altogether 65 (17 children and 48 adults) of the 367 patients at T1DM onset were positive for just one autoantibody. Isolated GAD-ab was most frequently found in adults (75.4%). In children, the occurrence of all three of the assessed antibodies was similar. One hundred and twenty-eight patients (82 children and 46 adults) were positive for two autoantibodies. IA2-ab and ZnT8-ab were the most frequent couple detected in children (53.6%) compared to adults (4.8%) (*p* < 0.0001). In adults, mostly GAD-ab with IA2-ab (53.6%) and GAD-ab with ZnT8-ab (41.4%) combinations were observed (Figs. 1, 2).

Both study groups demonstrated diverse autoantibodies pattern. In particular, 177 (81.1%) children were positive for ZnT8-ab, 176 children (80.7%) for IA2-ab and 152 (69.7%) for GAD-ab. IAA were tested exclusively in children and found in 128 individuals (58.7%) (girls: 58; boys: 42), in 30 (24%) children aged 0–4 years, 45 (35%) children aged 5–9 years, 40 (31%) aged 10–14 years and 13 (10%) patients aged 15–18 years.

On the contrary, only 52 adults (34.8%) were positive for ZnT8-ab, 65 (44.0%) for IA2-ab and 111 (74.8%) for GAD-ab, while 105 adult patients tested ICA positive (70.5%). Therefore, the presence of ZnT8-ab (81.1 vs. 34.8%, *p* < 0.0001) and IA2-ab (80.7 vs. 44%, *p* < 0.0001) was more common in children than in adults. Moreover, significantly higher titers of ZnT8-ab [407.7 (35.5–524.5) vs. 6.6 (0.0–157.9) U/ml, *p* < 0.0001] were found among pediatric population. In contrast, GAD-ab titers [3.9 (0.5–17.7) vs. 105.4 (8.9–545.8) U/ml, *p* < 0.0001] and IA2-ab titers [6.5 (1.5–19.3) vs. 17.8 (7.0–111.7) U/ml, *p* < 0.0001] were higher in adults compared to children (Table 1).

We found a positive correlation between age and the titer of GAD-ab (*R*_s_ − 0.296 *p* < 0.001) and a negative with the titer of ZnT8-ab (*R*_s_ − 0.314 *p* < 0.001) in both studied groups, while lower level of IA2-ab was correlated with increasing age in adults (*R*_s_ − 0.339 *p* < 0.001).

Seventy-eight new T1DM cases were complicated by DKA (mild: *n* = 23, moderate: *n* = 36, severe: *n* = 19). DKA was mainly observed in children (79.5%). Of the IAA positives children, 37 developed DKA, and this was moderate in 16, mild in 11 and severe in 11. ZnT8-ab (79.4 vs. 57.7%, *p* = 0.002) and IA2-ab (78.2 vs. 61.2%, *p* = 0.008) were detected more frequently in DKA group. Interestingly, the titers of IA2-ab [6.0 (2.5–24.2) vs. 11.0 (2.7–24.4) U/ml, *p* < 0.0001] and GAD-ab [4.7 (0.4–34.5) vs. 9.9 (2.2–105.4) U/ml, *p* < 0.00001] were lower in individuals with DKA (Table 2). There was a significant correlation between the number of positive antibodies and the severity of DKA (*R*_s_ 0.129 *p* = 0.014). In multiple regression analysis, the presence of positive ZnT8-ab was associated with higher risk of DKA independent of gender, agegroup (children and adults) and the number of autoantibodies (0–3) [OR = 2.44 (95% CI 1.0–5.94), *p* = 0.04].

**Table 2:** Characteristic of patients with and without DKA at type 1 diabetes onset

Variable	DKA (*n* = 78)	Without DKA (*n* = 289)	*p* value
Age (year) [IQR]	9 (5–14)	16 (9–32)	*p* < 0.001
ZnT8-ab (%)	79.4	57.7	*p* = 0.002
ZnT8-ab titer (U/ml)	250.1 (35.6–524.5)	110.5 (3.7–524.5)	*p* = 0.031
IA2-ab (%)	78.2	61.2	*p* = 0.008
IA2-ab titer (U/ml)	6.0 (2.5–24.2)	11.0 (2.7–24.4)	NS
GAD-ab (%)	62.8	72.6	NS
GAD-ab titer (U/ml)	4.7 (0.4–34.5)	9.9 (2.2–105.4)	*p* = 0.004

Data are expressed as medians; unless otherwise stated

*IQR* interquartile range, *NS* non-significant

## Discussion

We confirmed that children had more severe symptoms with lower β-cell function and more frequent DKA at disease presentation. Moreover, children and adults display diverse autoantibody patterns and severity of the autoimmune aggression, as estimated by the number of autoantibody found in both groups. These findings suggest that despite plausible influence of similar environmental factors, the intensity of the autoimmune attack may vary according to age.

### Prevalence of autoantibodies in age subgroups

Children had higher occurrence of all the three of the assessed antibodies (49.5%), whereas single antibody was rarely detected (7.8%). Adults were mostly positive for just 1 or 2 antibodies, and only 18.1% of them had three autoantibodies. This may suggest different intensity of β-cell autoimmunity. In subjects with only one autoantibody, isolated GAD-ab were the most common antibody found in adults, while in children the occurrence of any of three assessed antibodies was similar. In those with 2 positive antibodies, the combination of IA2-ZnT8-abs was the most common in children, but rare in adults. In adults, the combination of GAD-IA2-abs and slightly less frequent GAD-ZnT8 abs were observed. Similarly, as reported by Salonen et al., children were mostly positive for multiple antibodies with predominance of paired ZnT8-ab and IA2-ab [23]. Such an observation might reflect a positive relationship between GAD-ab occurrence and mild form of T1DM onset in adults. The majority of Chinese adults with LADA had only one positive autoantibody (85.9%), and the most detected single autoantibody was GAD-ab (67%) [24]. However, our analysis was performed on Caucasian ethnic group.

We observed higher prevalence and titer of ZnT8-ab in children (81.1%) than in adults (34.8%). Additionally, higher prevalence of IA-2-ab, but surprisingly lower titer of IA-2-ab and GAD-ab, was found in children compared to adults. In contrast, adults had mostly GAD-ab, with higher titer of those autoantibodies. Moreover, a lower prevalence of GAD-ab and a more aggressive diabetes onset in younger children might suggest that with an increase in T1DM prevalence not only a clinical phenotype has changed, but also immunophenotype reflected by a change of GAD-ab occurrence with time. Previously, Long et al. [25] reported the rise of IA2-ab and ZnT8-ab prevalence, while GAD-ab and IAA remained stable.

In our study, the prevalence of ZnT8-ab and other autoantibodies in adults was in concordance with that found in previous studies [26–29]. Compared to these findings in Caucasian populations using internationally validated assays, other studies had found an increased prevalence of ZnT8-ab in LADA only in patients with a Japanese ethnic background (19%) or when the cumulative reactivity to alternative ZnT8 constructs was considered [7, 30]. In children, we found more than 80% positivity for ZnT8-ab and IA2-ab, but less for GAD-ab. This proportion is slightly different from data reported by the Finnish Pediatric Diabetes Register, where children had mostly IA2-ab (76%), followed by GAD-ab (67%) and ZnT8-ab (62.7%) [23]. Głowińska-Olszewska et al. [31] noticed high prevalence of ZnT8-ab (72%) in Polish children with T1DM duration shorter than 5 years. This finding demonstrates that ZnT8-ab could identify heterogeneity in the age of diabetes onset and are useful markers of childhood onset T1DM. In the Italian multicenter study, the same sensitivity of ZnT8-ab and GAD-ab at diabetes diagnosis was observed in children [32]. Moreover, ZnT8-ab-positive individuals were younger, while GAD-ab positive older at diagnosis [31]. Those observations justify introduction of the three screen assay which detects ZnT8-ab, IA2-ab and GAD-ab, as a useful first steep screening of the β-cell autoimmunity in children [33].

### The diagnostic value of ZnT8-ab

There is a question about the diagnostic value of ZnT8-ab in adults. Mainly, if highest prevalence of ZnT8-ab was observed in children from 3 years old up to late adolescence, a clear tendency for gradual decline in ZnT8-ab was demonstrated thereafter. However, ZnT8-ab are highly β-cells specific, exclusively expressed in insulin containing secretory granules of β-cells, and it is a target of the autoimmune process [20, 34]. This high specificity emphasizes a diagnostic value of ZnT8-ab among adults mainly in patients with low-titer or absent GAD-ab or in acute onset of T1DM [26]. Particularly, we showed that addition of ZnT8-ab to GAD-ab and IA-2-ab measurements could improve the diagnostic accuracy of autoimmune diabetes in adults, but should be obligatory in children.

### Predictive role of autoantibodies in T1DM

In children, an aggressive autoimmune process may result in overt disease within a few months after the appearance of autoantibodies, whereas in older subjects the preclinical phase may continue for several years. The majority of children had IAA and GAD-ab as their first autoantibodies detectable in preclinical phase [35]. We demonstrated that children had very low titer of GAD-ab compared to adults. Based on our results, it might be also hypothesized that the level of GAD-ab may drop significantly at diagnosis. While, in adults, autoantibodies persisting long after diagnosis are usually high-titer GAD-ab, and much less frequently ZnT8-ab or IA2-ab [36]. Similarly, Sosenko et al. [37] indicated that in children GAD-ab titer tend to decline at diagnosis. ZnT8-ab positivity identified subjects at a higher risk of symptomatic diabetes. ZnT8 is located within β-cells secretory granules, and ZnT8-ab expression may not occur until there is enough β-cells damage to make this autoantigen perceptible for the immune system. Overall, the number and titer of autoantibodies decline after progression to T1DM. The type of persistent antibodies can be influenced by age at onset and could add information about clinical phenotypes. Perhaps, ZnT8-ab and GAD-ab might be considered as predictive markers for T1DM development, if they were measured in prediabetes phase. It seems that GAD-ab are more useful in such a situation, because they are positive prior to a mild autoimmune diabetes onset. However, in children with potentially short prediabetes phase, the predictive value of ZnT8-ab is not excluded. As, ZnT8-ab might be one of the key players in mediating both insulin secretion and β-cells mass in T1DM [38].

### Relationship between DKA and autoantibodies pattern

We confirmed that children are at greater risk of DKA at T1DM diagnosis. Yet, factors involved in DKA development and reasons why children are more prone to be diagnosed with DKA are unclear. DKA is either a consequence of delayed diagnosis, or it reflects intensity of destruction of β-cells. Nevertheless, it is proved that the occurrence of DKA has impact on long-term clinical course, probably through some deleterious influence on lower residual β-cell function and frequency of remission [39–42]. In our study, subjects with DKA were more likely to be positive for ZnT8-ab and IA-2-ab, but their titer of IA-2-ab and GAD-ab were lower. However, we found a correlation between the number of positive antibodies and the severity of DKA. Furthermore, ZnT8-ab positivity was associated with a higher risk of DKA independent of age and the number of autoantibodies. The association of ZnT8-ab and DKA has been analyzed before with conflicting results. Salonen et al. described that DKA at diagnosis in young children was less common among ZnT8-ab-positive subjects. In concordance with previous studies, ZnT8-ab was uncommon among children with the age of diabetes onset below 5 years. There was a steady increase in proportion of ZnT8-ab positivity in patients older than 5 years and a gradual decrease after 10 years of age [23]. Another study in Turkish children showed a high ZnT8-ab prevalence at T1DM onset (58%), but this was not associated with DKA [43]. However, our results are consistent with those in the Childhood Diabetes in Finland Study Group, in which positivity for ZnT8-ab was associated with older age and more frequent DKA in children at diagnosis as well as lower serum C-peptide concentrations and higher insulin doses over time compared to ZnT8-ab-negative peers [44]. Likewise, Kawasaki et al. [45] have shown that about two-thirds of adult-onset patients had the slow onset form, and some 20% present DKA at diagnosis. ZnT8-ab was identified in 58% patients with acute-onset and in 20% with slow-onset T1DM among the Japanese.

Overall in our study, ZnT8-ab, IA2-ab and GAD-ab were found in the majority of children, while GAD-ab was the main antibody presented in adults at diagnosis. ZnT8-ab emerged as an important marker for DKA at T1DM onset.

## Conclusions

Children are more prone to develop DKA, and they have lower concentrations of C-peptide at T1DM onset. Consequently, children feature worse residual β-cell function at diagnosis, which may negatively affect the prognosis of subsequent partial remission. Children and adults demonstrate distinct autoantibody patterns at the autoimmune diabetes diagnosis. ZnT8-ab and IA2-ab are more prevalent in children, while adults often display higher titer of GAD-ab. Our findings suggest that ZnT8-ab are associated with a more acute onset of T1DM due to a more aggressive autoimmune attack.

## Electronic supplementary material

Below is the link to the electronic supplementary material.

Supplementary material 1 (DOC 37 kb)

## Abbreviations

T1DM: Type 1 diabetes mellitus
DKA: Diabetes ketoacidosis
GAD-ab: Glutamic acid decarboxylase autoantibodies
IA2-ab: Protein tyrosine phosphatase autoantibodies
IAA: Insulin autoantibodies
ICA: Islet-cell antibodies
LADA: Latent autoimmune diabetes of adults
ZnT8-ab: Autoantibodies to zinc transporter-8
HbA1c: Glycated hemoglobin
