# Supplementary material for: Autoantibodies against zinc transporter 8 are related to age and metabolic state in patients with newly diagnosed autoimmune diabetes
Source: Acta Diabetol. 2018 Jan 11;55(3):287–94. doi: 10.1007/s00592-017-1091-x (PMC5829102; doi:10.1007/s00592-017-1091-x)
Supplement: Supplementary file 1 — Supplementary material 1 (DOC 37 kb) [file 592_2017_1091_MOESM1_ESM.doc]

Figure 3. Levels of GADA-ab, IA2-an and ZnT8-ab titres at T1DM onset in children and adults.
